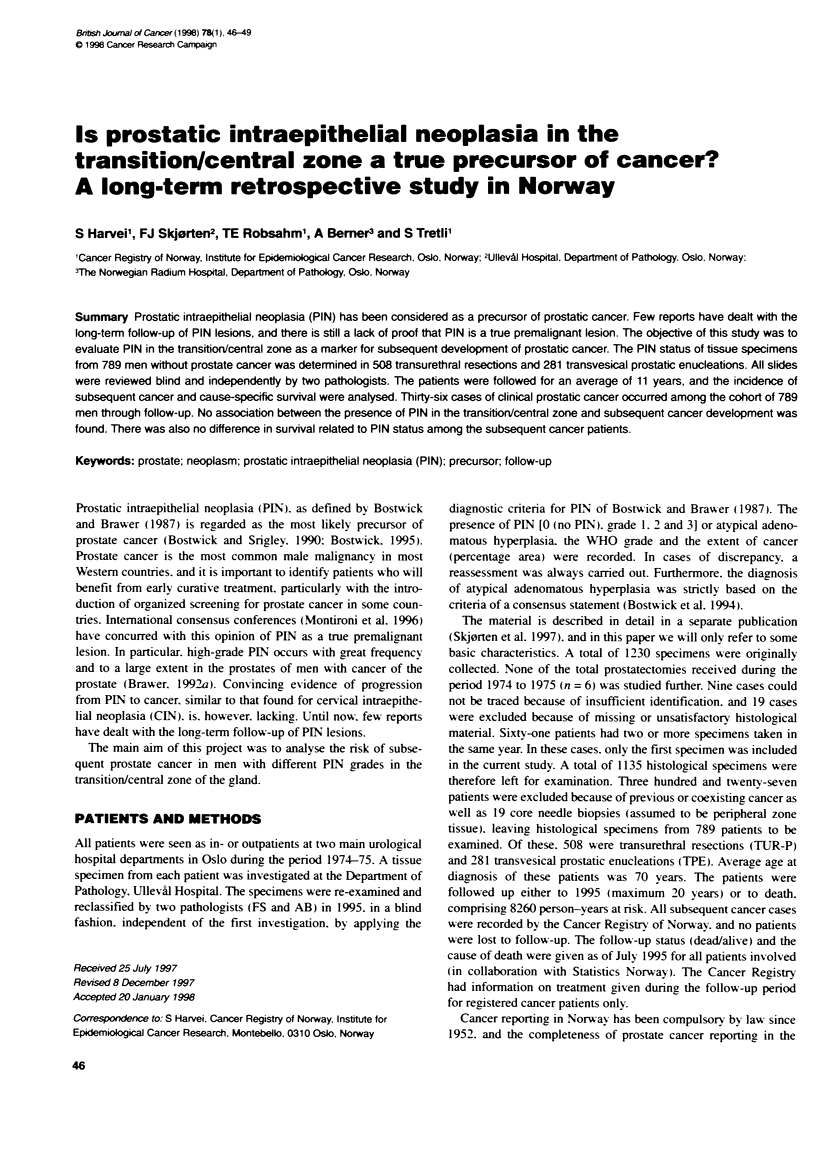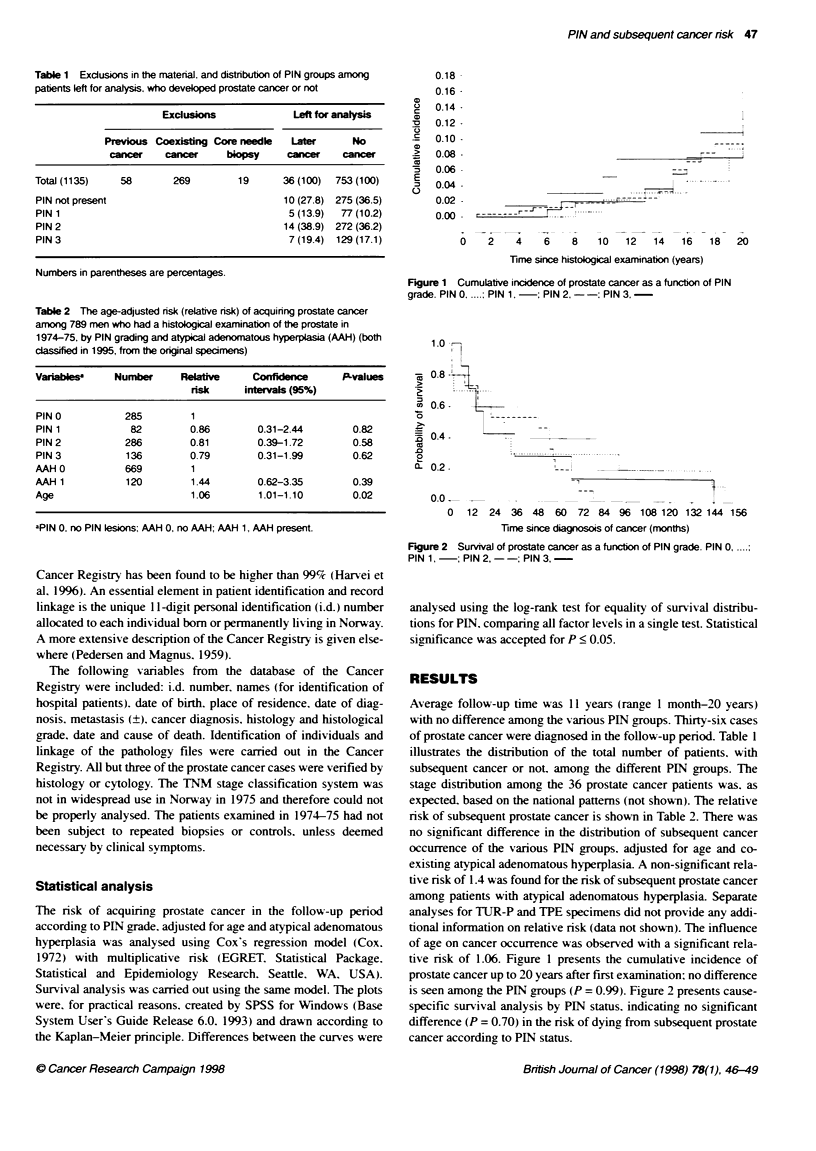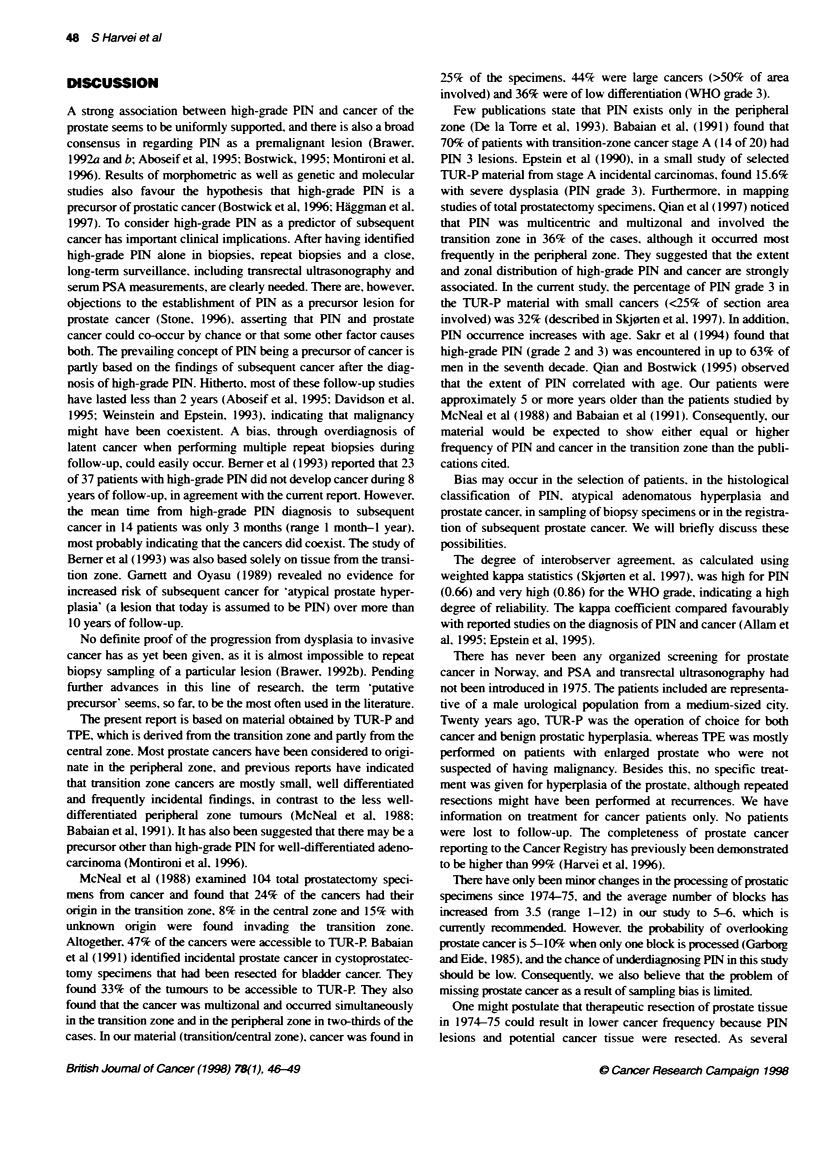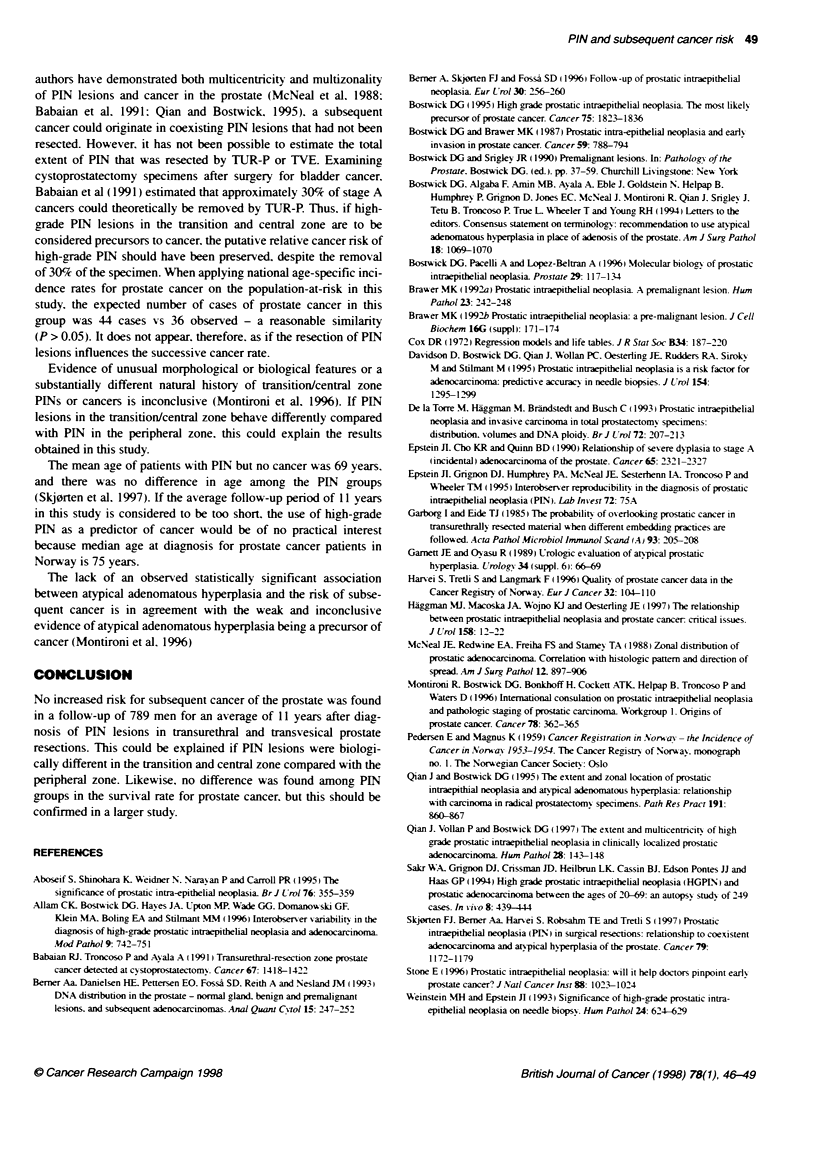# Is prostatic intraepithelial neoplasia in the transition/central zone a true precursor of cancer? A long-term retrospective study in Norway.

**DOI:** 10.1038/bjc.1998.440

**Published:** 1998-07

**Authors:** S. Harvei, F. J. Skjørten, T. E. Robsahm, A. Berner, S. Tretli

**Affiliations:** Cancer Registry of Norway, Institute for Epidemiological Cancer Research, Oslo.

## Abstract

Prostatic intraepithelial neoplasia (PIN) has been considered as a precursor of prostatic cancer. Few reports have dealt with the long-term follow-up of PIN lesions, and there is still a lack of proof that PIN is a true premalignant lesion. The objective of this study was to evaluate PIN in the transition/central zone as a marker for subsequent development of prostatic cancer. The PIN status of tissue specimens from 789 men without prostate cancer was determined in 508 transurethral resections and 281 transvesical prostatic enucleations. All slides were reviewed blind and independently by two pathologists. The patients were followed for an average of 11 years, and the incidence of subsequent cancer and cause-specific survival were analysed. Thirty-six cases of clinical prostatic cancer occurred among the cohort of 789 men through follow-up. No association between the presence of PIN in the transition/central zone and subsequent cancer development was found. There was also no difference in survival related to PIN status among the subsequent cancer patients.


British Journal of Cancer (1998) 78(1), 46–49
© 1998 Cancer Research Campaign

### Is prostatic intraepithelial neoplasia in the transition/central zone a true precursor of cancer? A long-term retrospective study in Norway

S Harvei[1], FJ Skjørten[2], TE Robsahm[1], A Berner[3] and S Tretli[1]

[1]Cancer Registry of Norway, Institute for Epidemiological Cancer Research, Oslo, Norway; [2]Ullevål Hospital, Department of Pathology, Oslo, Norway; [3]The Norwegian Radium Hospital, Department of Pathology, Oslo, Norway

**Summary** Prostatic intraepithelial neoplasia (PIN) has been considered as a precursor of prostatic cancer. Few reports have dealt with the long-term follow-up of PIN lesions, and there is still a lack of proof that PIN is a true premalignant lesion. The objective of this study was to evaluate PIN in the transition/central zone as a marker for subsequent development of prostatic cancer. The PIN status of tissue specimens from 789 men without prostate cancer was determined in 508 transurethral resections and 281 transvesical prostatic enucleations. All slides were reviewed blind and independently by two pathologists. The patients were followed for an average of 11 years, and the incidence of subsequent cancer and cause-specific survival were analysed. Thirty-six cases of clinical prostatic cancer occurred among the cohort of 789 men through follow-up. No association between the presence of PIN in the transition/central zone and subsequent cancer development was found. There was also no difference in survival related to PIN status among the subsequent cancer patients.

**Keywords:** prostate; neoplasm; prostatic intraepithelial neoplasia (PIN); precursor; follow-up

Prostatic intraepithelial neoplasia (PIN), as defined by Bostwick and Brawer (1987) is regarded as the most likely precursor of prostate cancer (Bostwick and Srigley, 1990; Bostwick, 1995). Prostate cancer is the most common male malignancy in most Western countries, and it is important to identify patients who will benefit from early curative treatment, particularly with the introduction of organized screening for prostate cancer in some countries. International consensus conferences (Montironi et al, 1996) have concurred with this opinion of PIN as a true premalignant lesion. In particular, high-grade PIN occurs with great frequency and to a large extent in the prostates of men with cancer of the prostate (Brawer, 1992*a*). Convincing evidence of progression from PIN to cancer, similar to that found for cervical intraepithelial neoplasia (CIN), is, however, lacking. Until now, few reports have dealt with the long-term follow-up of PIN lesions.

The main aim of this project was to analyse the risk of subsequent prostate cancer in men with different PIN grades in the transition/central zone of the gland.

### PATIENTS AND METHODS

All patients were seen as in- or outpatients at two main urological hospital departments in Oslo during the period 1974–75. A tissue specimen from each patient was investigated at the Department of Pathology, Ullevål Hospital. The specimens were re-examined and reclassified by two pathologists (FS and AB) in 1995, in a blind fashion, independent of the first investigation, by applying the diagnostic criteria for PIN of Bostwick and Brawer (1987). The presence of PIN [0 (no PIN), grade 1, 2 and 3] or atypical adenomatous hyperplasia, the WHO grade and the extent of cancer (percentage area) were recorded. In cases of discrepancy, a reassessment was always carried out. Furthermore, the diagnosis of atypical adenomatous hyperplasia was strictly based on the criteria of a consensus statement (Bostwick et al, 1994).

The material is described in detail in a separate publication (Skjørten et al, 1997), and in this paper we will only refer to some basic characteristics. A total of 1230 specimens were originally collected. None of the total prostatectomies received during the period 1974 to 1975 ($n = 6$) was studied further. Nine cases could not be traced because of insufficient identification, and 19 cases were excluded because of missing or unsatisfactory histological material. Sixty-one patients had two or more specimens taken in the same year. In these cases, only the first specimen was included in the current study. A total of 1135 histological specimens were therefore left for examination. Three hundred and twenty-seven patients were excluded because of previous or coexisting cancer as well as 19 core needle biopsies (assumed to be peripheral zone tissue), leaving histological specimens from 789 patients to be examined. Of these, 508 were transurethral resections (TUR-P) and 281 transvesical prostatic enucleations (TPE). Average age at diagnosis of these patients was 70 years. The patients were followed up either to 1995 (maximum 20 years) or to death, comprising 8260 person–years at risk. All subsequent cancer cases were recorded by the Cancer Registry of Norway, and no patients were lost to follow-up. The follow-up status (dead/alive) and the cause of death were given as of July 1995 for all patients involved (in collaboration with Statistics Norway). The Cancer Registry had information on treatment given during the follow-up period for registered cancer patients only.

Cancer reporting in Norway has been compulsory by law since 1952, and the completeness of prostate cancer reporting in the

*Received 25 July 1997*
*Revised 8 December 1997*
*Accepted 20 January 1998*

*Correspondence to:* S Harvei, Cancer Registry of Norway, Institute for Epidemiological Cancer Research, Montebello, 0310 Oslo, Norway

**Table 1** Exclusions in the material, and distribution of PIN groups among patients left for analysis, who developed prostate cancer or not

| | Exclusions | | | Left for analysis | |
|---|---|---|---|---|---|
| | Previous cancer | Coexisting cancer | Core needle biopsy | Later cancer | No cancer |
| Total (1135) | 58 | 269 | 19 | 36 (100) | 753 (100) |
| PIN not present | | | | 10 (27.8) | 275 (36.5) |
| PIN 1 | | | | 5 (13.9) | 77 (10.2) |
| PIN 2 | | | | 14 (38.9) | 272 (36.2) |
| PIN 3 | | | | 7 (19.4) | 129 (17.1) |

Numbers in parentheses are percentages.

**Table 2** The age-adjusted risk (relative risk) of acquiring prostate cancer among 789 men who had a histological examination of the prostate in 1974–75, by PIN grading and atypical adenomatous hyperplasia (AAH) (both classified in 1995, from the original specimens)

| Variables[a] | Number | Relative risk | Confidence intervals (95%) | *P*-values |
|---|---|---|---|---|
| PIN 0 | 285 | 1 | | |
| PIN 1 | 82 | 0.86 | 0.31–2.44 | 0.82 |
| PIN 2 | 286 | 0.81 | 0.39–1.72 | 0.58 |
| PIN 3 | 136 | 0.79 | 0.31–1.99 | 0.62 |
| AAH 0 | 669 | 1 | | |
| AAH 1 | 120 | 1.44 | 0.62–3.35 | 0.39 |
| Age | | 1.06 | 1.01–1.10 | 0.02 |

[a]PIN 0, no PIN lesions; AAH 0, no AAH; AAH 1, AAH present.

Cancer Registry has been found to be higher than 99% (Harvei et al, 1996). An essential element in patient identification and record linkage is the unique 11-digit personal identification (i.d.) number allocated to each individual born or permanently living in Norway. A more extensive description of the Cancer Registry is given elsewhere (Pedersen and Magnus, 1959).

The following variables from the database of the Cancer Registry were included: i.d. number, names (for identification of hospital patients), date of birth, place of residence, date of diagnosis, metastasis (±), cancer diagnosis, histology and histological grade, date and cause of death. Identification of individuals and linkage of the pathology files were carried out in the Cancer Registry. All but three of the prostate cancer cases were verified by histology or cytology. The TNM stage classification system was not in widespread use in Norway in 1975 and therefore could not be properly analysed. The patients examined in 1974–75 had not been subject to repeated biopsies or controls, unless deemed necessary by clinical symptoms.

### Statistical analysis

The risk of acquiring prostate cancer in the follow-up period according to PIN grade, adjusted for age and atypical adenomatous hyperplasia was analysed using Cox's regression model (Cox, 1972) with multiplicative risk (EGRET, Statistical Package, Statistical and Epidemiology Research, Seattle, WA, USA). Survival analysis was carried out using the same model. The plots were, for practical reasons, created by SPSS for Windows (Base System User's Guide Release 6.0, 1993) and drawn according to the Kaplan–Meier principle. Differences between the curves were analysed using the log-rank test for equality of survival distributions for PIN, comparing all factor levels in a single test. Statistical significance was accepted for $P \leq 0.05$.

**Figure 1** Cumulative incidence of prostate cancer as a function of PIN grade. PIN 0, ....; PIN 1, ——; PIN 2, — —; PIN 3, ——

**Figure 2** Survival of prostate cancer as a function of PIN grade. PIN 0, ....; PIN 1, ——; PIN 2, — —; PIN 3, ——

### RESULTS

Average follow-up time was 11 years (range 1 month–20 years) with no difference among the various PIN groups. Thirty-six cases of prostate cancer were diagnosed in the follow-up period. Table 1 illustrates the distribution of the total number of patients, with subsequent cancer or not, among the different PIN groups. The stage distribution among the 36 prostate cancer patients was, as expected, based on the national patterns (not shown). The relative risk of subsequent prostate cancer is shown in Table 2. There was no significant difference in the distribution of subsequent cancer occurrence of the various PIN groups, adjusted for age and co-existing atypical adenomatous hyperplasia. A non-significant relative risk of 1.4 was found for the risk of subsequent prostate cancer among patients with atypical adenomatous hyperplasia. Separate analyses for TUR-P and TPE specimens did not provide any additional information on relative risk (data not shown). The influence of age on cancer occurrence was observed with a significant relative risk of 1.06. Figure 1 presents the cumulative incidence of prostate cancer up to 20 years after first examination; no difference is seen among the PIN groups ($P = 0.99$). Figure 2 presents cause-specific survival analysis by PIN status, indicating no significant difference ($P = 0.70$) in the risk of dying from subsequent prostate cancer according to PIN status.

© *Cancer Research Campaign 1998*

### DISCUSSION

A strong association between high-grade PIN and cancer of the prostate seems to be uniformly supported, and there is also a broad consensus in regarding PIN as a premalignant lesion (Brawer, 1992*a* and *b*; Aboseif et al, 1995; Bostwick, 1995; Montironi et al, 1996). Results of morphometric as well as genetic and molecular studies also favour the hypothesis that high-grade PIN is a precursor of prostatic cancer (Bostwick et al, 1996; Häggman et al, 1997). To consider high-grade PIN as a predictor of subsequent cancer has important clinical implications. After having identified high-grade PIN alone in biopsies, repeat biopsies and a close, long-term surveillance, including transrectal ultrasonography and serum PSA measurements, are clearly needed. There are, however, objections to the establishment of PIN as a precursor lesion for prostate cancer (Stone, 1996), asserting that PIN and prostate cancer could co-occur by chance or that some other factor causes both. The prevailing concept of PIN being a precursor of cancer is partly based on the findings of subsequent cancer after the diagnosis of high-grade PIN. Hitherto, most of these follow-up studies have lasted less than 2 years (Aboseif et al, 1995; Davidson et al, 1995; Weinstein and Epstein, 1993), indicating that malignancy might have been coexistent. A bias, through overdiagnosis of latent cancer when performing multiple repeat biopsies during follow-up, could easily occur. Berner et al (1993) reported that 23 of 37 patients with high-grade PIN did not develop cancer during 8 years of follow-up, in agreement with the current report. However, the mean time from high-grade PIN diagnosis to subsequent cancer in 14 patients was only 3 months (range 1 month–1 year), most probably indicating that the cancers did coexist. The study of Berner et al (1993) was also based solely on tissue from the transition zone. Garnett and Oyasu (1989) revealed no evidence for increased risk of subsequent cancer for 'atypical prostate hyperplasia' (a lesion that today is assumed to be PIN) over more than 10 years of follow-up.

No definite proof of the progression from dysplasia to invasive cancer has as yet been given, as it is almost impossible to repeat biopsy sampling of a particular lesion (Brawer, 1992b). Pending further advances in this line of research, the term 'putative precursor' seems, so far, to be the most often used in the literature.

The present report is based on material obtained by TUR-P and TPE, which is derived from the transition zone and partly from the central zone. Most prostate cancers have been considered to originate in the peripheral zone, and previous reports have indicated that transition zone cancers are mostly small, well differentiated and frequently incidental findings, in contrast to the less well-differentiated peripheral zone tumours (McNeal et al, 1988; Babaian et al, 1991). It has also been suggested that there may be a precursor other than high-grade PIN for well-differentiated adenocarcinoma (Montironi et al, 1996).

McNeal et al (1988) examined 104 total prostatectomy specimens from cancer and found that 24% of the cancers had their origin in the transition zone, 8% in the central zone and 15% with unknown origin were found invading the transition zone. Altogether, 47% of the cancers were accessible to TUR-P. Babaian et al (1991) identified incidental prostate cancer in cystoprostatectomy specimens that had been resected for bladder cancer. They found 33% of the tumours to be accessible to TUR-P. They also found that the cancer was multizonal and occurred simultaneously in the transition zone and in the peripheral zone in two-thirds of the cases. In our material (transition/central zone), cancer was found in 25% of the specimens, 44% were large cancers (>50% of area involved) and 36% were of low differentiation (WHO grade 3).

Few publications state that PIN exists only in the peripheral zone (De la Torre et al, 1993). Babaian et al, (1991) found that 70% of patients with transition-zone cancer stage A (14 of 20) had PIN 3 lesions. Epstein et al (1990), in a small study of selected TUR-P material from stage A incidental carcinomas, found 15.6% with severe dysplasia (PIN grade 3). Furthermore, in mapping studies of total prostatectomy specimens, Qian et al (1997) noticed that PIN was multicentric and multizonal and involved the transition zone in 36% of the cases, although it occurred most frequently in the peripheral zone. They suggested that the extent and zonal distribution of high-grade PIN and cancer are strongly associated. In the current study, the percentage of PIN grade 3 in the TUR-P material with small cancers (<25% of section area involved) was 32% (described in Skjørten et al, 1997). In addition, PIN occurrence increases with age. Sakr et al (1994) found that high-grade PIN (grade 2 and 3) was encountered in up to 63% of men in the seventh decade. Qian and Bostwick (1995) observed that the extent of PIN correlated with age. Our patients were approximately 5 or more years older than the patients studied by McNeal et al (1988) and Babaian et al (1991). Consequently, our material would be expected to show either equal or higher frequency of PIN and cancer in the transition zone than the publications cited.

Bias may occur in the selection of patients, in the histological classification of PIN, atypical adenomatous hyperplasia and prostate cancer, in sampling of biopsy specimens or in the registration of subsequent prostate cancer. We will briefly discuss these possibilities.

The degree of interobserver agreement, as calculated using weighted kappa statistics (Skjørten et al, 1997), was high for PIN (0.66) and very high (0.86) for the WHO grade, indicating a high degree of reliability. The kappa coefficient compared favourably with reported studies on the diagnosis of PIN and cancer (Allam et al, 1995; Epstein et al, 1995).

There has never been any organized screening for prostate cancer in Norway, and PSA and transrectal ultrasonography had not been introduced in 1975. The patients included are representative of a male urological population from a medium-sized city. Twenty years ago, TUR-P was the operation of choice for both cancer and benign prostatic hyperplasia, whereas TPE was mostly performed on patients with enlarged prostate who were not suspected of having malignancy. Besides this, no specific treatment was given for hyperplasia of the prostate, although repeated resections might have been performed at recurrences. We have information on treatment for cancer patients only. No patients were lost to follow-up. The completeness of prostate cancer reporting to the Cancer Registry has previously been demonstrated to be higher than 99% (Harvei et al, 1996).

There have only been minor changes in the processing of prostatic specimens since 1974–75, and the average number of blocks has increased from 3.5 (range 1–12) in our study to 5–6, which is currently recommended. However, the probability of overlooking prostate cancer is 5–10% when only one block is processed (Garborg and Eide, 1985), and the chance of underdiagnosing PIN in this study should be low. Consequently, we also believe that the problem of missing prostate cancer as a result of sampling bias is limited.

One might postulate that therapeutic resection of prostate tissue in 1974–75 could result in lower cancer frequency because PIN lesions and potential cancer tissue were resected. As several

© Cancer Research Campaign 1998

authors have demonstrated both multicentricity and multizonality of PIN lesions and cancer in the prostate (McNeal et al, 1988; Babaian et al, 1991; Qian and Bostwick, 1995), a subsequent cancer could originate in coexisting PIN lesions that had not been resected. However, it has not been possible to estimate the total extent of PIN that was resected by TUR-P or TVE. Examining cystoprostatectomy specimens after surgery for bladder cancer, Babaian et al (1991) estimated that approximately 30% of stage A cancers could theoretically be removed by TUR-P. Thus, if high-grade PIN lesions in the transition and central zone are to be considered precursors to cancer, the putative relative cancer risk of high-grade PIN should have been preserved, despite the removal of 30% of the specimen. When applying national age-specific incidence rates for prostate cancer on the population-at-risk in this study, the expected number of cases of prostate cancer in this group was 44 cases vs 36 observed – a reasonable similarity ($P > 0.05$). It does not appear, therefore, as if the resection of PIN lesions influences the successive cancer rate.

Evidence of unusual morphological or biological features or a substantially different natural history of transition/central zone PINs or cancers is inconclusive (Montironi et al, 1996). If PIN lesions in the transition/central zone behave differently compared with PIN in the peripheral zone, this could explain the results obtained in this study.

The mean age of patients with PIN but no cancer was 69 years, and there was no difference in age among the PIN groups (Skjørten et al, 1997). If the average follow-up period of 11 years in this study is considered to be too short, the use of high-grade PIN as a predictor of cancer would be of no practical interest because median age at diagnosis for prostate cancer patients in Norway is 75 years.

The lack of an observed statistically significant association between atypical adenomatous hyperplasia and the risk of subsequent cancer is in agreement with the weak and inconclusive evidence of atypical adenomatous hyperplasia being a precursor of cancer (Montironi et al, 1996)

### CONCLUSION

No increased risk for subsequent cancer of the prostate was found in a follow-up of 789 men for an average of 11 years after diagnosis of PIN lesions in transurethral and transvesical prostate resections. This could be explained if PIN lesions were biologically different in the transition and central zone compared with the peripheral zone. Likewise, no difference was found among PIN groups in the survival rate for prostate cancer, but this should be confirmed in a larger study.

### REFERENCES

Aboseif S, Shinohara K, Weidner N, Narayan P and Carroll PR (1995) The significance of prostatic intra-epithelial neoplasia. *Br J Urol* **76**: 355–359

Allam CK, Bostwick DG, Hayes JA, Upton MP, Wade GG, Domanowski GF, Klein MA, Boling EA and Stilmant MM (1996) Interobserver variability in the diagnosis of high-grade prostatic intraepithelial neoplasia and adenocarcinoma. *Mod Pathol* **9**: 742–751

Babaian RJ, Troncoso P and Ayala A (1991) Transurethral-resection zone prostate cancer detected at cystoprostatectomy. *Cancer* **67**: 1418–1422

Berner Aa, Danielsen HE, Pettersen EO, Fosså SD, Reith A and Nesland JM (1993) DNA distribution in the prostate – normal gland, benign and premalignant lesions, and subsequent adenocarcinomas. *Anal Quant Cytol* **15**: 247–252

Berner A, Skjørten FJ and Fosså SD (1996) Follow-up of prostatic intraepithelial neoplasia. *Eur Urol* **30**: 256–260

Bostwick DG (1995) High grade prostatic intraepithelial neoplasia. The most likely precursor of prostate cancer. *Cancer* **75**: 1823–1836

Bostwick DG and Brawer MK (1987) Prostatic intra-epithelial neoplasia and early invasion in prostate cancer. *Cancer* **59**: 788–794

Bostwick DG and Srigley JR (1990) Premalignant lesions. In: *Pathology of the Prostate*, Bostwick DG. (ed.). pp. 37–59. Churchill Livingstone: New York

Bostwick DG, Algaba F, Amin MB, Ayala A, Eble J, Goldstein N, Helpap B, Humphrey P, Grignon D, Jones EC, McNeal J, Montironi R, Qian J, Srigley J, Tetu B, Troncoso P, True L, Wheeler T and Young RH (1994) Letters to the editors. Consensus statement on terminology: recommendation to use atypical adenomatous hyperplasia in place of adenosis of the prostate. *Am J Surg Pathol* **18**: 1069–1070

Bostwick DG, Pacelli A and Lopez-Beltran A (1996) Molecular biology of prostatic intraepithelial neoplasia. *Prostate* **29**: 117–134

Brawer MK (1992*a*) Prostatic intraepithelial neoplasia. A premalignant lesion. *Hum Pathol* **23**: 242–248

Brawer MK (1992*b* Prostatic intraepithelial neoplasia: a pre-malignant lesion. *J Cell Biochem* **16G** (suppl): 171–174

Cox DR (1972) Regression models and life tables. *J R Stat Soc* **B34**: 187–220

Davidson D, Bostwick DG, Qian J, Wollan PC, Oesterling JE, Rudders RA, Siroky M and Stilmant M (1995) Prostatic intraepithelial neoplasia is a risk factor for adenocarcinoma: predictive accuracy in needle biopsies. *J Urol* **154**: 1295–1299

De la Torre M, Häggman M, Brändstedt and Busch C (1993) Prostatic intraepithelial neoplasia and invasive carcinoma in total prostatectomy specimens: distribution, volumes and DNA ploidy. *Br J Urol* **72**: 207–213

Epstein JI, Cho KR and Quinn BD (1990) Relationship of severe dyplasia to stage A (incidental) adenocarcinoma of the prostate. *Cancer* **65**: 2321–2327

Epstein JI, Grignon DJ, Humphrey PA, McNeal JE, Sesterhenn IA, Troncoso P and Wheeler TM (1995) Interobserver reproducibility in the diagnosis of prostatic intraepithelial neoplasia (PIN). *Lab Invest* **72**: 75A

Garborg I and Eide TJ (1985) The probability of overlooking prostatic cancer in transurethrally resected material when different embedding practices are followed. *Acta Pathol Microbiol Immunol Scand (A)* **93**: 205–208

Garnett JE and Oyasu R (1989) Urologic evaluation of atypical prostatic hyperplasia. *Urology* **34** (suppl. 6): 66–69

Harvei S, Tretli S and Langmark F (1996) Quality of prostate cancer data in the Cancer Registry of Norway. *Eur J Cancer* **32**: 104–110

Häggman MJ, Macoska JA, Wojno KJ and Oesterling JE (1997) The relationship between prostatic intraepithelial neoplasia and prostate cancer: critical issues. *J Urol* **158**: 12–22

McNeal JE, Redwine EA, Freiha FS and Stamey TA (1988) Zonal distribution of prostatic adenocarcinoma. Correlation with histologic pattern and direction of spread. *Am J Surg Pathol* **12**, 897–906

Montironi R, Bostwick DG, Bonkhoff H, Cockett ATK, Helpap B, Troncoso P and Waters D (1996) International consulation on prostatic intraepithelial neoplasia and pathologic staging of prostatic carcinoma. Workgroup 1. Origins of prostate cancer. *Cancer* **78**: 362–365

Pedersen E and Magnus K (1959) *Cancer Registration in Norway – the Incidence of Cancer in Norway 1953–1954*. The Cancer Registry of Norway, monograph no. 1. The Norwegian Cancer Society: Oslo

Qian J and Bostwick DG (1995) The extent and zonal location of prostatic intraepithial neoplasia and atypical adenomatous hyperplasia: relationship with carcinoma in radical prostatectomy specimens. *Path Res Pract* **191**: 860–867

Qian J, Vollan P and Bostwick DG (1997) The extent and multicentricity of high grade prostatic intraepithelial neoplasia in clinically localized prostatic adenocarcinoma. *Hum Pathol* **28**: 143–148

Sakr WA, Grignon DJ, Crissman JD, Heilbrun LK, Cassin BJ, Edson Pontes JJ and Haas GP (1994) High grade prostatic intraepithelial neoplasia (HGPIN) and prostatic adenocarcinoma between the ages of 20–69: an autopsy study of 249 cases. *In vivo* **8**: 439–444

Skjørten FJ, Berner Aa, Harvei S, Robsahm TE and Tretli S (1997) Prostatic intraepithelial neoplasia (PIN) in surgical resections: relationship to coexistent adenocarcinoma and atypical hyperplasia of the prostate. *Cancer* **79**: 1172–1179

Stone E (1996) Prostatic intraepithelial neoplasia: will it help doctors pinpoint early prostate cancer? *J Natl Cancer Inst* **88**: 1023–1024

Weinstein MH and Epstein JI (1993) Significance of high-grade prostatic intraepithelial neoplasia on needle biopsy. *Hum Pathol* **24**: 624–629

© Cancer Research Campaign 1998